# Identification of NKX2-5 and GATA4 sequence variations in patients with cardiac septation defects

**DOI:** 10.1186/1755-8166-7-S1-P27

**Published:** 2014-01-21

**Authors:** Yashvanthi Borkar, K Ranjan Shetty, K Krishnanand, Rajasekhar Moka

**Affiliations:** 1 Department of Biotechnology, School of Life Sciences, Manipal University, Manipal, India; 2 Department of Cardiology, KMC, Manipal University, Manipal, India

## Background

Cardiac septation defects (CHD) are the problems with the structure of the heart during the development and is one of the most common among congenital heart defect (CHD) which occurs ~1% in general population. The etiology for the majority of these defects may be multifactorial. However, advancement in understanding the molecular basis of normal heart development has revealed the necessity of numerous genes which are predominantly expressed during the heart morphogenesis. Many cohort studies have shown that cardiac transcription factors NKX2-5, GATA4 and TBX5 are the master regulators during heart development. The mutations in any of these genes results in the failure of normal development of heart leading to septal defects. The study was carried out to identify the nucleotide sequence variation in the patients with Atrial Septal Defects (ASD), Ventricular Septal Defects (VSD) and Atrioventricular Septal Defect (AVSD).

## Materials and methods

Fifty seven patients with ASD (n=51) and VSD (n=6) were included in the study after a thorough evaluation of Echocardiography and clinical phenotypes. All the coding regions and flanking introns of NKX2-5 and GATA4 which are predominantly expressed in the heart were amplified by polymerase chain reaction and sequenced using ABI PRISM 3130 automated sequencer.

## Results

No patient was found to have NKX2-5 and GATA4 mutations, however single nucleotide polymorphism (c.63A>G; rs2277923) in NKX2-5 was observed in 20 patients with Ostium secundum ASD.

## Conclusions

Since the mutation frequency is very low (NKX2-5 ~1-4%; GATA4 ~1%) in these cases, we need to study more number of patients to get expected frequency.

